# Supplementary material for: Assessing the link between hygienic material use during menstruation and self-reported reproductive tract infections among women in India: a propensity score matching approach
Source: PeerJ. 2023 Nov 17;11:e16430. doi: 10.7717/peerj.16430 (PMC10658888; doi:10.7717/peerj.16430)
Supplement: Appendix S1 [file peerj-11-16430-s001.docx]

**Appendix 1: Propensity Score Matching (PSM)**

This study used the PSM technique to evaluate the impact of hygienic materials use on RTIs among women in India. The decision to use PSM was made because the women who used hygienic materials were not randomly selected. As a result, selection bias may have been introduced into the estimated treatment effect.

When randomized control trials, widely considered the gold standard for measuring the effectiveness of treatments and interventions on outcomes, are not feasible, PSM can be a helpful tool for evaluating the treatment effect using cross-sectional, observational, or non-experimental data. For example, in PSM, the average outcome for women who used hygienic materials is compared (treated group) to that for women who did not use hygienic materials (control group) while controlling for observed differences between the two groups.

By utilizing PSM in this study, we created a matched sample of women with similar observable characteristics but different exposures to hygienic materials. This allowed us to compare the average RTI prevalence rates between these two groups and estimate the impact of hygienic materials on RTI incidence while controlling for potential confounding variables.

Overall, the use of PSM in this study enhanced the validity and accuracy of our findings and provided valuable insights into the impact of hygienic materials on RTI prevalence among women in India.

We employed propensity score matching since our treatment variable was binary, with two values: 1 for treated and 0 for control. PSM model, a probit/logit model, was used with the equation (Hanck et al., 2023)-

Φ (α + β₁X₁ + β₂X₂ + ... + βₖXₖ + ϵ) = Pr (DTreat = 1)

ln (Pr (DTreat = 1) / (1 – Pr (DTreat = 1))) = α + β₁X₁ + β₂X₂ + ... + βₖXₖ + ϵ

Where:

Φ represents the cumulative distribution function of the standard normal distribution.

ln is the natural logarithm.

DTreat is a binary treatment indicator (1 if treated, 0 if not treated).

X₁, X₂, …, Xₖ are control variables that influence the treatment assignment.

Α is the intercept.

Β₁, β₂, …, βₖ are the coefficients associated with the control variables.

ϵ represents the error term.

In this study, we employed a nearest neighbor matching method with a caliper of 0.2 standard deviations of the logit of the propensity score, to match propensity scores between treatment and control groups (Rosenbaum & Rubin, 1985; Caliendo & Kopeinig, 2005; Austin, 2011; Greifer, 2022). The nearest neighbor matching method involved iteratively matching treatment units with the nearest available control unit that met the test's criteria.

***Average treatment effect (ATE), the average treatment effect on treated (ATT), and the average treatment effect on untreated (ATU)***

The treatment effect for individual I is calculated as

Δ = y1i - y0i

where y1 is the value of y if the individual received treatment and y0 if the individual did not.

Our study has two groups: the treatment and control groups. The average treatment effect (ATE) of the treatment and control groups in our study can be calculated as follows:

$$ATE=E[y1i- y0i]=\frac{1}{N}\sum_{i=0}^{N} \left( y1i- y0i \right)$$

The average treatment effect on the treated (ATT) was calculated using the counterfactual model. The ATT examines the impact of hygienic materials use during menstruation on those women who have used hygienic materials:

ATT=E(YI|D=1)-E(Y0|D=1)

Where E(YI|D=1) is the average number of women who have reported RTIs (D=1) symptoms and have used hygienic materials, and. E(Y0|D=1) is the counterfactual outcome; it is not observable and needs to be estimated.

Another measure is the average treatment effect in the untreated (ATU), which measures the impact that the use of hygienic materials would have had on the women who have not used hygienic materials:

𝐴𝑇𝑈 = 𝐸(𝑌1 |𝐷 = 0)−𝐸(𝑌0|𝐷 = 0)

where 𝐸(𝑌1 |𝐷 = 0) is the average number of women using hygienic materials but not having symptoms of RTIs. 𝐸(𝑌0|𝐷 = 0) is the counterfactual outcome, which is unobserved.

**Validation of estimates**

***Common support***

To assess the quality of matching and ensure appropriate balance of covariates between the treated and untreated groups, we used the *pstest* command in Stata 16. As the matching was based on propensity scores, it was crucial to verify the existence of similarities between the observations in the treated group and the control group. This verification aligns with the concept of common support, where the overlap of propensity score distributions in both groups indicates the region within which valid matching can occur (Dixit, Dwivedi & Ram, 2013; Singh, 2016; Binci et al., 2018). Observations falling within this common support region are considered "on support," as their propensity scores make them suitable for meaningful matching. Conversely, observations outside this common support region, known as "off support," are excluded from the matching process due to the lack of comparable counterparts in the opposing group.

***Balancing test***

To test the quality of balance between the treated and control groups, we conducted a comprehensive analysis. Firstly, we quantified the pre-matching and post-matching bias (in %), for all matching variables. Subsequently, we calculated the reduction in bias (in %) after the matching. Criteria for successful matching were based upon two key conditions: First, if, post-matching, the bias for all matching variables is below the 10% threshold (Singh, 2016). Second, after matching, a substantial reduction in bias for matching variables, which should be visible in the graph.

Moreover, we examined the variance ratios of the matching variables, comparing the ratio of variances between women from treated group and women from control group, before and after the matching process. The formula is:

Variance ratio=$\frac{S^{2} of treated group}{S^{2} of control group}$; where, S^2^ is respective groups’ variance.

If there is perfect balance across samples, then covariates should be distributed equally and hence this ratio should be equal to one. Hence, after matching, if the variance ratios for each matching variable approaches to 1, this indicates a favourable balance between the control and treated groups (Zhang et al., 2019).

***Significance of the model***

The study also used pseudo R2 and the log likelihood ratio tests obtained using *pstest* command in the *psmatch2* package of Stata16, which assesses the overall significance of the model used in the matching analysis.

At first, we calculated the pseudo R^2^ before and after matching, and compared the pseudo R^2^’s before and after matching. “After matching there should be no systematic differences in the distribution of covariates between treated and control groups and therefore, the pseudo-R^2^ should be fairly low” (Caliendo & Kopeinig, 2005). Furthermore, we calculated the mean and median bias of all variables before and after matching to determine whether the matching procedure had successfully reduced the bias in our dataset (Dixit, Dwivedi & Ram, 2013; Dixit, Gupta & Dwivedi, 2018).

We also computed Rubin’s B and Rubin’s R, as additional measures to ensure our quality of matching. Rubin’s B reflects the absolute standardised difference of the means of the propensity score in the treated and control groups (Binci et al., 2018). Rubin’s R is the ratio of the treated to control variances of the propensity scores. The Rubin’s B should be less than 25 and R should be between the range of 0.5 to 2 (Singh, 2016; Mak & Fancourt, 2019).

**Sensitivity analysis**

The PSM method cannot fully control for unobservable factors. As a result, it is critical to determine if unobserved variables might influence treatment effect inferences, which may invalidate our model. As a result, matching estimations must be supported with sensitivity analysis. Mantel-Haenszel bounds proposed by Becker (2007), which provide upper and lower bound estimates of significance levels at a given level of hidden bias, were used as a sensitivity analysis in our study (Becker & Caliendo, 2007).

**REFERENCES**

Austin PC. 2011. Optimal caliper widths for propensity-score matching when estimating differences in means and differences in proportions in observational studies. *Pharmaceutical Statistics* 10:150–161. DOI: 10.1002/pst.433.

Becker S, Caliendo M. 2007. mhbounds - Sensitivity Analysis for Average Treatment Effects. *Pagina 24* 6054.

Binci M, Hebbar M, Jasper P, Rawle G. 2018. Matching, differencing on repeat: Propensity score matching and Methodological guidance and an repeated cross-sectional data: difference-in-differences with empirical application in education. *Oxford Policy Management*.

Caliendo M, Kopeinig S. 2005. Some Practical Guidance for the Implementation of Propensity Score Matching.

Dixit P, Dwivedi LK, Ram F. 2013. Strategies to improve child immunization via antenatal care visits in India: A propensity score matching analysis. *PloS one* 8:1–10. DOI: 10.1371/journal.pone.0066175.

Dixit P, Gupta A, Dwivedi LK. 2018. Impact Evaluation of Integrated Child Development Services in Rural India : Propensity Score Matching Analysis. DOI: 10.1177/2158244018785713.

Greifer N. 2022. Matching Methods. *Impact Evaluation in International Development: Theory, Methods, and Practice*:205–231. DOI: 10.1596/978-1-4648-1497-6_ch13.

Hanck C, Arnold M, Gerber A, Schmelzer M. 2023. Probit and Logit Regression. In: *Introduction to Econometrics with R*.

Mak HW, Fancourt D. 2019. Arts engagement and self-esteem in children: results from a propensity score matching analysis. *Annals of the New York Academy of Sciences* 1449:36–45. DOI: 10.1111/nyas.14056.

Rosenbaum PR, Rubin DB. 1985. Constructing a control group using multivariate matched sampling methods that incorporate the propensity score. *American Statistician* 39:33–38. DOI: 10.1080/00031305.1985.10479383.

Singh A. 2016. Availability and inequality in the distribution of health workers in the public health system in rural India. University of Portsmouth.

Zhang Z, Kim HJ, Lonjon G, Zhu Y. 2019. Balance diagnostics after propensity score matching. *Annals of Translational Medicine* 7:16–16. DOI: 10.21037/atm.2018.12.10.
