# Supplementary material for: Assessing the link between hygienic material use during menstruation and self-reported reproductive tract infections among women in India: a propensity score matching approach
Source: PeerJ. 2023 Nov 17;11:e16430. doi: 10.7717/peerj.16430 (PMC10658888; doi:10.7717/peerj.16430)
Supplement: Appendix S2 [file peerj-11-16430-s002.docx]

**Appendix 2: Percentage of sampled women reporting symptoms of RTI and use of hygienic materials by background characteristics, NFHS 2019-21**

| **Background characteristics** | **Prevalence of RTI (weighted %)** | **95% CI [Lower, Upper]** | **Use of unhygienic materials** | **95% CI**  **[Lower, Upper]** | **Use of hygienic materials** | **95% CI**  **[Lower, Upper]** |
| --- | --- | --- | --- | --- | --- | --- |
| **Age (in years)** |  |  |  |  |  |  |
| 15-19 | 1.48 | [1.24,1.77] | 47.41 | [45.93,48.89] | 52.59 | [51.11,54.07] |
| 20-24 | 6.63 | [6.04,7.29] | 47.95 | [46.53,49.38] | 52.05 | [50.62,53.47] |
| **Age at menarche (in years)** | | | | | | |
| ≤12 | 3.95 | [3.14,4.96] | 46.57 | [44.17,48.98] | 53.43 | [51.02,55.83] |
| 13-15 | 4.03 | [3.66,4.43] | 48.17 | [46.90,49.44] | 51.83 | [50.56,53.10] |
| ≥16 | 3.58 | [2.60,4.93] | 42.62 | [38.47,46.88] | 57.38 | [53.12,61.53] |
| **Years of schooling** |  |  |  |  |  |  |
| No education | 9.78 | [7.98,11.94] | 82.56 | [79.54,85.22] | 17.44 | [14.78,20.46] |
| 1-5 years | 7.06 | [5.37,9.24] | 75.90 | [72.79,78.75] | 24.10 | [21.25,27.21] |
| 6-10 years | 4.11 | [3.60,4.68] | 53.42 | [51.85,54.98] | 46.58 | [45.02,48.15] |
| 11 years and above | 2.97 | [2.58,3.41] | 35.91 | [34.48,37.35] | 64.09 | [62.65,65.52] |
| **Social groups** |  |  |  |  |  |  |
| SC | 4.39 | [3.66,5.27] | 49.75 | [47.58,51.92] | 50.25 | [48.08,52.42] |
| ST | 3.65 | [2.92,4.55] | 58.35 | [55.28,61.36] | 41.65 | [38.64,44.72] |
| OBC | 3.98 | [3.54,4.48] | 49.35 | [47.73,50.97] | 50.65 | [49.03,52.27] |
| Other | 3.73 | [3.00,4.62] | 36.37 | [34.20,38.59] | 63.63 | [61.41,65.80] |
| **Religion** |  |  |  |  |  |  |
| Hindu | 3.83 | [3.47,4.23] | 47.55 | [46.27,48.83] | 52.45 | [51.17,53.73] |
| Muslim | 4.91 | [3.94,6.09] | 54.52 | [51.41,57.59] | 45.48 | [42.41,48.59] |
| Christian | 3.37 | [2.06,5.48] | 39.63 | [33.77,45.81] | 60.37 | [54.19,66.23] |
| Others | 5.17 | [3.71,7.17] | 26.23 | [22.40,30.46] | 73.77 | [69.54,77.60] |
| **Wealth quintile** |  |  |  |  |  |  |
| Poorest | 4.59 | [3.75,5.59] | 75.76 | [73.93,77.49] | 24.24 | [22.51,26.07] |
| Poorer | 4.40 | [3.70,5.22] | 60.68 | [58.75,62.59] | 39.32 | [37.41,41.25] |
| Middle | 4.50 | [3.76,5.37] | 47.17 | [45.11,49.23] | 52.83 | [50.77,54.89] |
| Richer | 3.32 | [2.75,4.02] | 35.98 | [33.86,38.15] | 64.02 | [61.85,66.14] |
| Richest | 3.22 | [2.61,3.96] | 22.61 | [20.74,24.60] | 77.39 | [75.40,79.26] |
| **Exposure to mass media** |  |  |  |  |  |  |
| No exposure to mass media | 5.30 | [4.50,6.24] | 70.91 | [68.53,73.18] | 29.09 | [26.82,31.47] |
| Exposed to any one kind of mass media | 3.75 | [3.39,4.14] | 43.22 | [42.00,44.45] | 56.78 | [55.55,58.00] |
| **Discussed MH with CHW in last 3 months** | | | | | | |
| No | 4.02 | [3.68,4.40] | 47.76 | [46.56,48.96] | 52.24 | [51.04,53.44] |
| Yes | 2.56 | [1.22,5.32] | 43.03 | [36.85,49.45] | 56.97 | [50.55,63.15] |
| **Currently working** |  |  |  |  |  |  |
| No | 3.84 | [3.48,4.23] | 46.96 | [45.71,48.22] | 53.04 | [51.78,54.29] |
| Yes | 4.93 | [4.07,5.95] | 51.89 | [49.50,54.28] | 48.11 | [45.72,50.50] |
| **Takes bath during menstruation** | | | | | | |
| No | 4.43 | [2.93,6.64] | 58.36 | [52.86,63.66] | 41.64 | [36.34,47.14] |
| Yes | 3.98 | [3.64,4.35] | 47.32 | [46.12,48.52] | 52.68 | [51.48,53.88] |
| **Consumption of alcohol** |  |  |  |  |  |  |
| No | 3.98 | [3.64,4.35] | 47.68 | [46.49,48.87] | 52.32 | [51.13,53.51] |
| Yes | 9.54 | [3.36,24.27] | 46.10 | [33.40,59.33] | 53.90 | [40.67,66.60] |
| **Place of residence** |  |  |  |  |  |  |
| Urban | 3.24 | [2.62,4.01] | 30.07 | [28.03,32.19] | 69.93 | [67.81,71.97] |
| Rural | 4.34 | [3.94,4.76] | 55.59 | [54.30,56.86] | 44.41 | [43.14,45.70] |
| **Region of residence** |  |  |  |  |  |  |
| North | 4.45 | [3.77,5.25] | 34.60 | [32.44,36.82] | 65.40 | [63.18,67.56] |
| Central | 4.62 | [3.98,5.36] | 68.30 | [66.53,70.02] | 31.70 | [29.98,33.47] |
| East | 4.74 | [3.83,5.85] | 54.02 | [51.57,56.45] | 45.98 | [43.55,48.43] |
| West | 2.63 | [1.97,3.49] | 33.29 | [29.73,37.06] | 66.71 | [62.94,70.27] |
| Southern | 2.79 | [2.21,3.52] | 28.23 | [25.82,30.77] | 71.77 | [69.23,74.18] |
| North-east | 3.51 | [2.74,4.49] | 59.28 | [55.76,62.70] | 40.72 | [37.30,44.24] |
| **Use of materials during menstruation** | | | | | | |
| Unhygienic | 4.92 | [4.42,5.48] |  |  |  |  |
| Hygienic | 3.15 | [2.76,3.60] |  |  |  |  |

Note: RTI= reproductive tract infections, CI= confidence intervals, SC= scheduled caste, ST= scheduled tribe, OBC= other backward classes, MH= menstrual hygiene, CHW= community health workers
