# Supplementary material for: Assessing the link between hygienic material use during menstruation and self-reported reproductive tract infections among women in India: a propensity score matching approach
Source: PeerJ. 2023 Nov 17;11:e16430. doi: 10.7717/peerj.16430 (PMC10658888; doi:10.7717/peerj.16430)
Supplement: Appendix S3 [file peerj-11-16430-s003.docx]

**Appendix 3: Post matching estimates (percentage bias, and variance ratios)**

| **Variable** | **Unmatched Matched** | **Mean** | | **% bias** | **% reduced bias** | **t test** | | **Variance ratio** |
| --- | --- | --- | --- | --- | --- | --- | --- | --- |
|  |  | **Treated** | **Control** |  |  | **t** | **p** |  |
| **Age (in years)** | U | 19.4 | 19.4 | -3.0 |  | -2.5 | <0.001 | 1.0 |
|  | M | 19.3 | 19.4 | -2.7 | 10.5 | -2.3 | 0.022 | 1.0 |
| **Age at menarche (in years)** | U | 13.5 | 13.4 | 4.7 |  | 3.9 | <0.001 | 1.2 |
|  | M | 13.5 | 13.4 | 1.7 | 64.1 | 1.4 | 0.172 | 1.0 |
| **Years of schooling** | U | 11.0 | 9.0 | 57.5 |  | 48.1 | <0.001 | 1.5 |
|  | M | 11.0 | 11.0 | -1.2 | 97.9 | -1.2 | 0.248 | 0.9 |
| **Social groups** | U | 0.4 | 0.4 | -12.3 |  | -10.3 | <0.001 | 1.0 |
|  | M | 0.4 | 0.4 | -1.3 | 89.4 | -1.1 | 0.271 | 1.0 |
| **Religion** | U | 0.8 | 0.8 | 0.3 |  | 0.3 | 0.795 | 1.1 |
|  | M | 0.8 | 0.8 | -0.2 | 23.7 | -0.2 | 0.842 | 1.0 |
| **Wealth factor score** | U | 410000.0 | -240000.0 | 74.2 |  | 62.1 | <0.001 | 0.9 |
|  | M | 400000.0 | 380000.0 | 2.7 | 96.3 | 2.3 | 0.019 | 1.0 |
| **Exposure to mass media** | U | 0.9 | 0.8 | 37.8 |  | 31.6 | <0.001 | 1.2 |
|  | M | 0.9 | 0.9 | -1.8 | 95.2 | -1.9 | 0.057 | 1.0 |
| **Discussed MH with CHW in last 3 months** | U | 0.0 | 0.0 | 2.6 |  | 2.2 | 0.027 | 0.6 |
|  | M | 0.0 | 0.0 | -1.1 | 59.0 | -0.9 | 0.396 | 1.0 |
| **Currently working** | U | 0.1 | 0.2 | -11.7 |  | -9.8 | <0.001 | 1.2 |
|  | M | 0.1 | 0.1 | -0.1 | 98.8 | -0.1 | 0.902 | 0.9 |
| **Takes bath during menstruation** | U | 1.0 | 0.9 | 9.9 |  | 8.3 | <0.001 | 0.9 |
|  | M | 1.0 | 1.0 | 0.1 | 99.3 | 0.1 | 0.950 | 1.0 |
| **Consumption of alcohol** | U | 0.1 | 0.1 | -8.9 |  | -7.5 | <0.001 | 3.0 |
|  | M | 0.1 | 0.1 | 1.4 | 84.2 | 1.3 | 0.200 | 1.0 |
| **Place of residence** | U | 0.0 | 0.0 | -1.5 |  | -1.2 | 0.215 | 2.3 |
|  | M | 0.0 | 0.0 | 0.1 | 94.6 | 0.1 | 0.944 | 1.0 |
| **Region of residence** |  |  |  |  |  |  |  |  |
| North | U | 1.7 | 1.9 | -42.9 |  | -35.8 | <0.001 | 0.8 |
|  | M | 1.7 | 1.7 | 1.2 | 97.3 | 0.9 | 0.388 | 0.9 |
| Central | U | 0.2 | 0.4 | -47.7 |  | -39.8 | <0.001 | 1.1 |
|  | M | 0.2 | 0.2 | 0.8 | 98.2 | 0.8 | 0.409 | 1.0 |
| East | U | 0.1 | 0.2 | -11.0 |  | -9.1 | <0.001 | 0.9 |
|  | M | 0.1 | 0.2 | -0.5 | 95.8 | -0.4 | 0.688 | 1.0 |
| West | U | 0.1 | 0.1 | 10.3 |  | 8.7 | <0.001 | 0.9 |
|  | M | 0.1 | 0.1 | 0.0 | 100.0 | 0.0 | 1.000 | 1.0 |
| Southern | U | 0.2 | 0.1 | 36.4 |  | 30.5 | <0.001 | 0.7 |
|  | M | 0.2 | 0.2 | -6.3 | 82.8 | -4.4 | 0.008 | 1.0 |
| North-east | U | 0.1 | 0.1 | -5.0 |  | -4.2 | <0.001 | 1.2 |
|  | M | 0.1 | 0.1 | -0.9 | 81.1 | -0.8 | 0.423 | 1.0 |
